# Supplementary material for: Public priorities on locally-driven sea level rise planning on the East Coast of the United States
Source: PeerJ. 2020 May 4;8:e9044. doi: 10.7717/peerj.9044 (PMC7204830; doi:10.7717/peerj.9044)
Supplement: Supplemental Information 2 — This file contains the full text of all questions and response meanings presented to survey respondents, as presented electronically during this survey. [file peerj-08-9044-s002.pdf]

Please read the consent form and provide your response below

INFORMED CONSENT FORM – Please print or save a copy of this page for your records

RESEARCH PROCEDURES
This research is being conducted to help identify priorities in local sea level rise planning in coastal communities in the eastern United States, and is open to adults who live in, work in, regularly visit, or are otherwise connected to these coastal communities. If you agree to participate, you will be asked to answer questions about your opinions on planning for sea level rise and flooding. No personally identifiable information is collected, although some non-identifiable demographic information is. The survey should take approximately 10 minutes to complete.

RISKS
There are no foreseeable risks for participating in this research.

BENEFITS
There are no benefits to you as a participant other than to further research in this subject.

CONFIDENTIALITY
is an anonymous survey: names and other personal identifiers will not be collected. While it is understood that no computer transmission can be perfectly secure, reasonable efforts will be made to protect the confidentiality of your transmission.

PARTICIPATION
Your participation is voluntary, and you may withdraw from the study at any time and for any reason. If you decide not to participate or if you withdraw from the study, there is no penalty or loss of benefits to which you are otherwise entitled. There are no costs to you or any other party. Compensation for participating in the study is determined by the survey company and may include points, rewards, or monetary compensation.

CONTACT
This research is being conducted by Adam Carpenter, a student at George Mason University in the Environmental Science and Policy department. He may be reached at 703-957-8823 for questions or to report a research-related problem, and his faculty advisor Dr. Robert Jonas can be reached at 703-993-1030. You may contact the George Mason University Institutional Review Board Office at 703-993-4121 if you have questions or comments regarding your rights as a participant in the research.

This research has been reviewed according to George Mason University procedures governing your participation in this research and has been assigned the study number 1168842-1.

CONSENT
I have read this form, all of my questions have been answered by the research staff, and by continuing, I agree to participate in this study.

I consent and agree to participate in this study

I do not consent and do not wish to participate

Do you live in, work in, or regularly visit a coastal community in the east coast of the United States?

- ☐ I work in a coastal community
- ☐ I live in a coastal community
- ☐ I regularly visit a coastal community
- ☐ *None of the above*

« Back | Continue »

Please state the importance of the following issues in your community

	Very Unimportant (1)	Somewhat Unimportant (2)	Neither Important nor Unimportant (3)	Somewhat Important (4)	Very Important (5)
Helping people with limited resources	○	○	○	○	○
Reducing taxes	○	○	○	○	○
Growing the economy	○	○	○	○	○
Protecting against future flooding	○	○	○	○	○
Preparing for climate change	○	○	○	○	○
Maintaining utilities and related infrastructure	○	○	○	○	○
Protecting the environment	○	○	○	○	○
Maintaining roads and other transportation infrastructure	○	○	○	○	○
Preparing for sea level rise	○	○	○	○	○
Protecting property from natural disasters	○	○	○	○	○

« Back Continue »

In your community, how important are the following components in preparing for future flooding and sea level rise?

	Very Unimportant (1)	Somewhat Unimportant (2)	Neither Important nor Unimportant(3)	Somewhat Important (4)	Very Important (5)
Developing maps and tools to learn where flooding will and won't likely cause damage	○	○	○	○	○
Educating the community on the causes of flooding and sea level rise	○	○	○	○	○
Calculating the most cost-effective places and things to protect	○	○	○	○	○
Building physical barriers (sea walls, levies, dunes, etc.) to protect against flooding	○	○	○	○	○
Preparing to respond and/or evacuate when flooding happens	○	○	○	○	○
Implementing required policies to reduce future flood damage	○	○	○	○	○
Working in the community to implement voluntary protections	○	○	○	○	○
Finding ways to postpone making changes until more research is done	○	○	○	○	○

« Back Continue »

Are there any other components to preparing for future flooding and sea level rise that are important? (Please state and rate 1-5. If none, write N/A)

1 is lowest (very unimportant) and 5 is highest (very important)

« Back Continue »

How vulnerable is your community to damage from the following hazards?

	Not at all Vulnerable (1)	Somewhat Vulnerable (2)	Vulnerable (3)	Highly Vulnerable (4)	Exceptionally Vulnerable (5)
Repeated flooding from high tides	○	○	○	○	○
Other types of natural disasters	○	○	○	○	○
Increased flooding if sea level rises in the future	○	○	○	○	○
Water surge damage from hurricanes and severe storms	○	○	○	○	○

« Back Continue »

Please rate how high a priority protecting each the following items in your community from flooding and future sea level rise should be

	Not at all a Priority (1)	Somewhat a Priority (2)	A Moderate Priority (3)	A High Priority (4)	An Exceptionally High priority (5)
Sewer / Wastewater collection and treatment					
Electric Power					
Natural wetlands, wildlife areas, etc					
Natural Gas / Heating fuel distribution					
Roads and highways					
Railroads, subways, busses & stations					
Beaches and similar coastal areas					
Drinking Water					
Individual homes and residences					
Houses of worship					
Parks and public spaces					
Government facilities (includes police & fire depts)					
Individual businesses, offices, shops, etc.					
Stormwater and Green Infrastructure					
Places of cultural importance					

« Back Continue »

Are there any other items in your community that should be priorities for protection? (Please state and rate 1-5. If none, write N/A)

1 is lowest (not at all a priority) and 5 is highest (an exceptionally high priority)

« Back

Continue »

Should preparing for future flooding and sea level rise be mostly private sector (and individual) responsibility, public sector (and government) responsibility or a mix of both?

- Entirely Private Sector
- Mostly Private Sector
- Equal Mix of Public and Private Sectors
- Mostly Public Sector
- Entirely Public Sector

« Back

How useful are the following methods in determining how much money should be spent protecting against floods and the effects of future sea level rise?

	Not at all Useful (1)	Somewhat Useful (2)	Useful (3)	Highly Useful (4)	Exceptionally Useful (5)
Encourage insurance companies to require upgrades on homes/businesses to reduce risks as a condition of insurance	○	○	○	○	○
Set policies to encourage individuals/businesses to pay for their own protection to minimize local government costs	○	○	○	○	○
Hold public meetings to identify highest priorities and vote on methods to pay for them	○	○	○	○	○
Increase funding by raising local fees for beaches and other amenities	○	○	○	○	○
Increase funding for protection by cutting other local programs and services	○	○	○	○	○
Increase funding by raising local sales taxes	○	○	○	○	○
Increase funding by raising local property taxes	○	○	○	○	○
Use only money already used for protection (no change)	○	○	○	○	○
Minimize use of local taxes but utilize state/federal money when available	○	○	○	○	○
Increase funding by raising local income taxes	○	○	○	○	○

« Back Continue »

Are there any other methods to determine how much money should be spent protecting against floods and the effects of future sea level rise? (Please state and rate 1-5. If none, write N/A)

1 is lowest (not at all useful) and 5 is highest (exceptionally useful)

« Back Continue »

No type of protection is foolproof. Advanced protections are more complicated and expensive, while basic protections will fail more often. How strong should your community make its flooding and sea level rise protection?

	Fails less than 10% of years (1 in 10 years average)	Fails less than 2.5% of years (1 in 50 years average)	Fails less than 1% of years (1 in 100 years average)	Fails less than 0.2% of years (1 in 500 years average	Fails less than 0.1% of years (1 in 1,000 years average)
Fails Causing Minor Flooding	○	○	○	○	○
Fails Causing Major Flooding	○	○	○	○	○

« Back Continue »

How helpful are the following techniques in resolving potential conflict in developing a plan to protect against flooding from sea level rise in your community?

	Not at all Helpful (1)	Somewhat Helpful (2)	Helpful (3)	Very Helpful (4)	Exceptionally Helpful (5)
Hold public meetings to identify ways to resolve conflicts	○	○	○	○	○
Perform cost and benefit analysis on various ways to move forward	○	○	○	○	○
Start with measures that have the greatest public support	○	○	○	○	○
Increase educational efforts through the media about the risks and impacts of flooding	○	○	○	○	○
Hold votes on options to resolve disputes	○	○	○	○	○
Discuss with preparedness experts about ways to improve protection against floods	○	○	○	○	○
Make some measures optional for individual homes and businesses	○	○	○	○	○
DIscuss with scientists about the chances and locations of future flooding	○	○	○	○	○

« Back   Continue »

Are there any other techniques to resolve potential conflict in your community? (Please state and rate 1-5. If none, write N/A)

1 is lowest (not at all helpful) and 5 is highest (exceptionally helpful)

« Back

Continue »

How appropriate are the following responses to protect against flooding and future sea level rise?

	Very Inappropriate (1)	Somewhat Inappropriate (2)	Neither Appropriate nor Inappropriate (3)	Somewhat Appropriate (4)	Very Appropriate (5)
Prevent new development on the most vulnerable areas	○	○	○	○	○
Develop and enhance man-made physical barriers (sea walls, levies, etc.)	○	○	○	○	○
Require new structures to be built at higher elevations	○	○	○	○	○
Harden public infrastructure (roads, utilities, etc.) against damage	○	○	○	○	○
Increase cost of insuring high-risk areas	○	○	○	○	○
Develop and enhance natural physical barriers (such as wetlands or sand dunes)	○	○	○	○	○
Raise the elevation of existing structures	○	○	○	○	○
Don’t provide assistance for areas at highest risk	○	○	○	○	○
Develop and enhance early warning systems to notify residents about upcoming floods	○	○	○	○	○
Remove existing development from the most vulnerable areas over time	○	○	○	○	○

« Back   Continue »

Are there any other responses to protect against flooding and future sea level rise in your community? (Please state and rate 1-5. If none, write N/A)

1 is lowest (very inappropriate) and 5 is highest (very appropriate)

« Back Continue »

Please indicate your annual household income

- Less than $25,000
- $25,000 to $34,999
- $35,000 to $49,999
- $50,000 to $74,999
- $75,000 to $99,999
- $100,000 to $149,999
- $150,000 to $199,999
- $200,0000 or more
- Decline to Answer

« Back

How much would you consider yourself an environmentalist?

- Not at all
- Somewhat
- Moderately
- Highly
- Exceptionally

« Back

Please indicate your primary occupation

If none, you may write student, retired, unemployed, etc.

« Back

Continue »

Please indicate your highest level of education completed

- No formal education
- 8th grade or less
- Some high school
- High school diploma
- Some college
- Bachelors degree
- Some graduate school
- Masters degree
- Doctoral or Professional Degree
- Decline to Answer

« Back

Please specify your ethnicity

White or Caucasian

Asian / Pacific Islander

Native American or American Indian

Black or African American

Hispanic or Latino

Multi-racial

Decline to Answer

Other: please specify Submit

« Back

What political party would you consider yourself most aligned with?

Not affiliated (independent)

Republican

Democratic

Another party

« Back

Was there any part of the survey you were confused about, or anything about flooding and sea level rise that the survey did not address but should have? (If none, write N/A)

« Back | Continue »
