# Supplementary material for: Public priorities on locally-driven sea level rise planning on the East Coast of the United States
Source: PeerJ. 2020 May 4;8:e9044. doi: 10.7717/peerj.9044 (PMC7204830; doi:10.7717/peerj.9044)
Supplement: Table S1 [file peerj-08-9044-s003.docx]

**Supplemental Tables**

Table S1. Summary statistics for community vulnerability surveyed sorted by mean score (Carpenter, 2019)

| **Vulnerability Type** | **Mean** | **Median** | **Mode** | **Standard Deviation** | **Number (Percent) Ranking 1 or 2** | **Number (Percent) Ranking 4 or 5** |
| --- | --- | --- | --- | --- | --- | --- |
| Water surge damage from hurricanes and severe storms | 3.35 | 3 | 4 | 1.261 | 137 (27.2%) | 248 (49.3%) |
| Increased flooding if sea level rises in the future | 3.17 | 3 | 4 | 1.299 | 254 (50.5%) | 223 (44.3%) |
| Other natural disasters | 3.12 | 3 | 3 | 1.082 | 154 (30.6%) | 180 (35.8%) |
| Repeated flooding from high tides | 2.89 | 3 | 2 | 1.351 | 220 (43.7%) | 187 (37.2%) |

Table S2. Distribution of responses on protection strength (Carpenter, 2019)

| **Potential Failure Rate** | **Major Flooding** | | **Minor Flooding** | |
| --- | --- | --- | --- | --- |
|  | **Total** | **Percent** | **Total** | **Percent** |
| Fails less than 0.1% of years (1 in 1,000 years average) | 58 | 11.5% | 35 | 7.0% |
| Fails less than 0.2% of years (1 in 500 years average) | 92 | 18.3% | 61 | 12.1% |
| Fails less than 1% of years (1 in 100 years average) | 180 | 35.8% | 168 | 33.4% |
| Fails less than 2.5% of years (1 in 50 years average) | 98 | 19.5% | 126 | 25.0% |
| Fails less than 10% of years (1 in 10 years average) | 75 | 14.9% | 113 | 22.5% |

Table S3. Respondent’s household income (Carpenter, 2019)

| **Amount** | **Frequency** | **Percentage** |
| --- | --- | --- |
| $200,000 or more | 21 | 4.2% |
| $150,000 to $199,999 | 20 | 4.0% |
| $100,000 to $149,999 | 64 | 12.7% |
| $75,000 to $99,999 | 79 | 15.7% |
| $50,000 to $74,999 | 107 | 21.3% |
| $35,000 to $49,999 | 79 | 15.7% |
| $25,000 to $34,999 | 51 | 10.1% |
| Less than $25,000 | 66 | 13.1% |
| **Total** | **503** | **100.0%** |

Table S4. Respondent’s self-rated environmentalism (Carpenter, 2019)

| **Environmentalist** | **Frequency** | **Percent** |
| --- | --- | --- |
| Exceptionally (5) | 43 | 8.5% |
| Highly (4) | 117 | 23.3% |
| Moderately (3) | 199 | 39.6% |
| Somewhat (2) | 115 | 22.9% |
| Not at all (1) | 29 | 5.8% |
| **Total** | **503** | **100.0%** |

Table S5. Respondent’s job titles (Carpenter, 2019)

| **Job Title/Category** | **Frequency** | **Percentage** |
| --- | --- | --- |
| President or Owner | 34 | 6.80% |
| C-Level Executive (CIO, CTO, COO, CMO, etc.) | 16 | 3.20% |
| Senior Vice President | 7 | 1.40% |
| Vice President | 3 | 0.60% |
| Director | 15 | 3.00% |
| Senior Manager | 14 | 2.80% |
| Manager | 82 | 16.30% |
| Analyst/Associate | 87 | 17.30% |
| Entry Level | 64 | 12.70% |
| Student | 68 | 13.50% |
| Retired | 113 | 22.50% |
| **Total** | **503** | **100.0%** |

Table S6. Respondent’s self-reported ethnicities (Carpenter, 2019)

| **Ethnicity** | **Frequency** | **Percentage** |
| --- | --- | --- |
| Asian / Pacific Islander | 21 | 4.2% |
| Black or African American | 48 | 9.5% |
| Decline to Answer | 4 | 0.8% |
| Hispanic or Latino | 25 | 5.0% |
| Multi-racial | 10 | 2.0% |
| Native American or American Indian | 4 | 0.8% |
| Other: Latino | 1 | 0.2% |
| Other: Mixed races | 1 | 0.2% |
| White or Caucasian | 389 | 77.3% |
| **Total** | **503** | **100.0%** |

Table S7. Respondent’s self-identified political parties (Carpenter, 2019)

| **Political Party** | **Frequency** | **Percent** |
| --- | --- | --- |
| Another party | 23 | 4.6% |
| Democratic | 214 | 42.5% |
| Not affiliated (independent) | 142 | 28.2% |
| Republican | 124 | 24.7% |
| **Total** | **503** | **100.0%** |

Table S8. Respondent’s age groups across respondents (Carpenter, 2019)

| **Age Group** | **Frequency** | **Percent** |
| --- | --- | --- |
| 18 to 24 | 80 | 15.9% |
| 25 to 34 | 133 | 26.4% |
| 35 to 44 | 98 | 19.5% |
| 45 to 54 | 72 | 14.3% |
| 55 to 64 | 55 | 10.9% |
| 65 and over | 65 | 12.9% |
| **Total** | **503** | **100%** |

Table S9. Respondent’s genders

| **Age Group** | **Frequency** | **Percent** |
| --- | --- | --- |
| Female | 335 | 66.6% |
| Male | 168 | 33.4% |

Table S10. Respondent’s locations across eastern coastal states (Carpenter, 2019)

| **State** | **Frequency** | **Percent** |
| --- | --- | --- |
| CT | 16 | 3.2% |
| DC | 7 | 1.4% |
| DE | 2 | 0.4% |
| FL | 108 | 21.5% |
| MA | 34 | 6.8% |
| MD | 18 | 3.6% |
| ME | 6 | 1.2% |
| NC | 59 | 11.7% |
| NH | 9 | 1.8% |
| NJ | 41 | 8.2% |
| NY | 96 | 19.1% |
| PA | 42 | 8.3% |
| RI | 5 | 1.0% |
| SC | 29 | 5.8% |
| VA | 31 | 6.2% |
| **Total** | **503** | **100.0%** |
